# Tribo-Dynamics of Dual-Star Planetary Gear Systems: Modeling, Analysis, and Experiments

**DOI:** 10.3390/s25154709

**Published:** 2025-07-30

**Authors:** Jiayu Zheng, Yonggang Xiang, Changzhao Liu, Yixin Wang, Zonghai Mou

**Affiliations:** 1 State Key Laboratory of Mechanical Transmission for Advanced Equipment, Chongqing University, Chongqing 400044, China; zhengjiayu@cqu.edu.cn (J.Z.); 30024228@alu.cqu.edu.cn (Y.X.); 202407021270t@stu.cqu.edu.cn (Y.W.); 2 School of Mechanical Engineering, Sichuan University of Light and Chemical Engineering, Yibin 644000, China; 323085503233@stu.suse.edu.cn

**Keywords:** dual-star planetary gear system, thermal elastohydrodynamic lubrication, tribo-dynamics, surrogate model, experimental validation

## Abstract

To address the unclear coupling mechanism between thermal elastohydrodynamic lubrication (TEHL) and dynamic behaviors in planetary gear systems, a novel tribo-dynamic model for dual-star planetary gears considering TEHL effects is proposed. In this model, a TEHL surrogate model is first established to determine the oil film thickness and sliding friction force along the tooth meshing line. Subsequently, the dynamic model of the dual-star planetary gear transmission system is developed through coordinate transformations of the dual-star gear train. Finally, by integrating lubrication effects into both time-varying mesh stiffness and time-varying backlash, a tribo-dynamic model for the dual-star planetary gear transmission system is established. The study reveals that the lubricant film thickness is positively correlated with relative sliding velocity but negatively correlated with unit line load. Under high-speed conditions, a thickened oil film induces premature meshing contact, leading to meshing impacts. In contrast, under high-torque conditions, tooth deformation dominates meshing force fluctuations while lubrication influence diminishes. By establishing a test bench for the planetary gear transmission system, the obtained simulation conclusions are verified. This research provides theoretical and experimental support for the design of high-reliability planetary gear systems.

## 1. Introduction

In planetary gear systems, dual-star gear systems are widely used across various industries due to their advantages, such as excellent torque balance, high transmission efficiency, and superior power density [1,2]. Under high-load, variable operating conditions, the vibration characteristics, lubrication performance, temperature field, and tooth failure mechanisms of dual-star gear systems have become key research focuses [3]. However, current gear contact methodologies predominantly rely on Hertzian contact theory or iso thermal elastohydrodynamic lubrication (TEHL) theory. These conventional approaches not only neglect the bidirectional coupling mechanism between TEHL effects and dynamic behavior but also fail to accurately characterize pressure-temperature field distortions induced by transient squeeze effects, ultimately resulting in significant discrepancies between simulated and actual operating conditions [4,5,6]. Therefore, developing a tribo-dynamics coupled model for planetary gears, incorporating TEHL contact characteristics—with experimental validation—becomes essential.

During the gear meshing process, the lubricant film undergoes deformation due to tooth compression, thereby altering the load distribution on tooth surfaces [7]. In studies of lubrication effects on tooth load distribution, Lu et al. [8,9] proposed a novel mesh stiffness model for spur gears considering TEHL contact, revealing that lubricated contact reduces tooth temperature and lowers meshing stiffness under light loads/high speeds, emphasizing the need to account for combined lubrication-thermal effects. Chimanpure et al. [10] established a transient mixed elastohydrodynamic lubrication (EHL) model for helical gears, incorporating real roughness and time-varying contact kinematics to assess lubrication behavior and power loss, with validation against experimental results. Wang et al. [11,12] presented an adhesive wear model for helical gears in mixed EHL, which revealed that lubricants reduce wear, while roughness and torque exacerbate it, and geometric/operational parameters like helix angle and speed mitigate wear. Xiao et al. [13,14] established a line-contact non-Newtonian transient TEHL damping model for spur gears, demonstrating that increased normal damping enhances resistance to meshing impacts and vibration suppression, while reduced tangential damping improves lubricant flow and mitigates frictional temperature rise. Pei et al. [15,16] introduced a reliability prediction method for gear EHL under stochastic torque inputs, identifying higher failure probabilities of full-film lubrication at pinion tooth roots and validating the consistency between boundary lubrication failure probabilities and actual gear wear states. Although these studies have deepened the understanding of gear lubrication contact mechanisms, obvious limitations remain in research on lubrication effects on the dynamic behavior of transmission systems [17].

Since the state of tooth lubrication has a significant effect on the dynamic behavior of gears, an increasing number of scholars have begun to consider their coupling effect [18,19]. In the study of planetary gearing lubrication, Huangfu et al. [20,21] proposed a dynamic pitting evolution model for planetary gear sets, integrating tribo-dynamic coupling, and revealed that mixed EHL and dynamic load distribution govern pitting topography, with gamma distributions better capturing involute-direction pitting than normal distributions. Jian et al. [22] proposed a TEHL dynamic model for planetary gear trains, revealing that increased modification coefficients can improve lubrication performance while exacerbating engagement impacts and reducing oil film stiffness. Ning et al. [23] investigated the dynamic characteristics of planetary gear mechanisms under mixed EHL; the results showed that lubrication conditions critically influence nonlinear dynamics, with operational parameters directly altering stiffness and system responses. Wu et al. [24] proposed a novel time-varying mesh stiffness (TVMS) model for planetary gear-annulus pairs integrating EHL, demonstrating improved accuracy over traditional potential energy methods and emphasizing the critical role of EHL dynamics in gear transmission systems. Wang et al. [25] established a comprehensive dynamic model for planetary gear systems considering mixed EHL friction and TVMS, showing through numerical analysis that surface friction accelerates the onset of chaos and creates richer bifurcation patterns, especially in high-frequency operating conditions. Although the above studies have carried out comprehensive research on the dynamic modeling and dynamic characteristics of planetary gears under lubrication, they are all theoretical studies, and research on the experimental aspects of planetary gear lubrication is significantly lacking. In addition, the current study mainly couples the TEHL model with the dynamic model in the form of oil film stiffness, ignoring the effect of oil film thickness on backlash [26,27,28].

In order to solve the above problems, a novel tribo-dynamic model of the dual-star planetary gear transmission system is proposed in this paper, which can accurately reflect the dynamic characteristics of the transmission system under different operating conditions, and its validity is experimentally verified. The innovations of this study are as follows:

1. A surrogate model for TEHL equations under different operating conditions is developed, which significantly improves the computational efficiency of the tribo-dynamic model.
2. A novel tribo-dynamics coupling method is achieved through oil film stiffness and time-varying backlash.
3. Critical lubrication effects on system dynamics are identified: Substantial influence under high-speed conditions but minimal impact under heavy loads.
4. A test bench for the planetary gear systems is established, experimentally validating the simulation results.

The content of this paper is organized as follows. In Section 2, a surrogate model for TEHL in dual-star planetary gears is developed. In Section 3, a tribo-dynamic model of the transmission system is established. In Section 4, the dynamic characteristics of the transmission system under different operating conditions are analyzed. In Section 5, the experimental validation of the proposed model is conducted.

## 2. Solution of TEHL Equations for Planetary Gear Systems

During gear meshing, the comprehensive radius of curvature, relative sliding speed, and other parameters vary along the meshing line, consequently altering the interfacial lubrication state. Therefore, it is necessary to carry out a kinematic analysis, and then the TEHL equations of the planetary gear transmission are solved. To enhance computational efficiency, a surrogate model is employed to replace the full TEHL numerical solution model.

### 2.1. Mathematical Model of Planetary Gear Transmission

**Figure 1:**
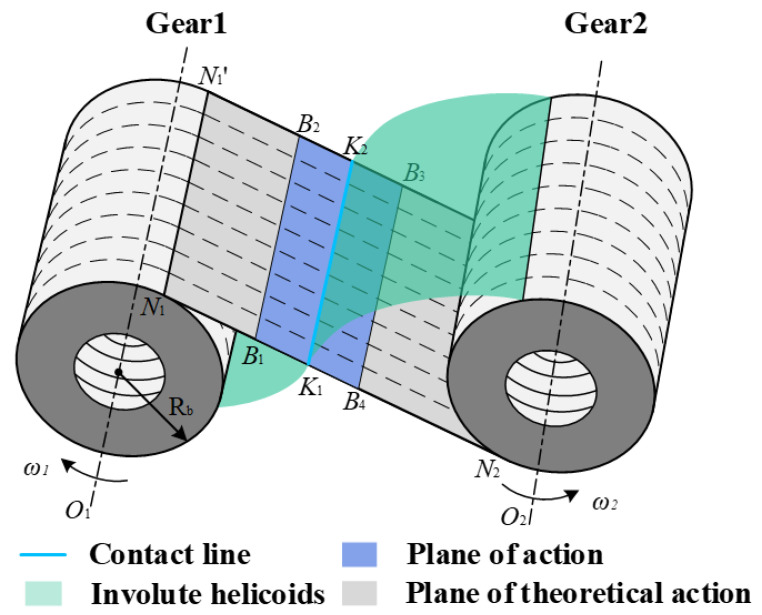
Transmission diagram of a straight cylindrical gear.

The meshing mode of the planetary gear is mainly divided into external meshing and internal meshing, and the external meshing of the gear is shown in Figure 1. In the figure, *O*_1_ and *O*_2_ indicate the rotary axis of the driving and driven gear, respectively; *ω*_1_ and *ω*_2_ indicate the rotational speed; *N*_1_*N*′_1_*N*′_2_*N*_2_ indicates the theoretical meshing plane of the two gears, *B*_1_*B*′_1_*B*′_2_*B*_2_ is the actual meshing plane, and *K*_1_*K*_2_ indicates the contact line.

The tooth contact of two external meshing gears can be equated to two cylindrical contacts with *N*_1_*N*′_1_, *N*′_2_*N*_2_ as the rotational axes and with opposite directions, respectively. The radius of rotation varies with the meshing line. Similarly, the internal meshing gears can be equated to two cylindrical contacts with the same rotational direction [1].

For the external mesh pair, the radius of curvature at the contact line *K*_1_*K*_2_ can be expressed as follows:

(1) $$\left\{\begin{array}{l} \rho_{tk1}=r_{b1}\tan\alpha_{k}-r_{b2}(\tan\alpha_{ta2}-\tan\alpha_{k})+s\\ \rho_{tk2}=r_{b2}\tan\alpha_{k}+r_{b2}(\tan\alpha_{ta2}-\tan\alpha_{k})-s \end{array}\right.$$

For the internal mesh pair, the radius of curvature at the contact line *K*_1_*K*_2_ is then expressed as follows:

(2) $$\left\{\begin{array}{l} \rho_{tk1}=r_{b1}\tan\alpha_{k}+r_{b2}(\tan\alpha_{ta2}-\tan\alpha_{k})+s\\ \rho_{tk2}=r_{b2}\tan\alpha_{k}+r_{b2}(\tan\alpha_{ta2}-\tan\alpha_{k})+s \end{array}\right.$$

where *ρ_tk_*_1_ and *ρ_tk_*_2_ are the radii of the curvature of the driving and driven gears, respectively. *r_bi_*, *α_tai_*, and *α_k_* are the radius of the base circle, the pressure angle of the addendum circle, and the meshing angles at the contact line *K*_1_*K*_2_ of the driving and driven gears, respectively. *s* represents the distance between the contact line *K*_1_*K*_2_ and the meshing starting end *B*_1_*B*′_1_ [1].

Therefore, the equivalent radius of curvature *R_k_*, relative sliding speed *u_k_*, and sliding-rolling ratio *SR_k_* at the contact line *K*_1_*K*_2_ can be expressed as follows:

(3) $$\left\{\begin{array}{l} R_{k}=\frac{\rho_{tk1}\rho_{tk2}}{\rho_{tk1}+\rho_{tk2}}\\ u_{k}=\frac{v_{tk1}+v_{tk2}}{2}\\ SR_{k}=\frac{2(v_{tk2}-v_{tk1})}{v_{tk1}+v_{tk2}} \end{array}\right.$$

where *v_tk_*_1_ and *v_tk_*_2_ represent the tangential direction speed of the driving and driven gears at the contact line *K*_1_*K*_2_, respectively, expressed as follows:

(4) $$\left\{\begin{array}{l} v_{tk1}=\omega_{1}\rho_{t1}\\ v_{tk2}=\omega_{2}\rho_{t2} \end{array}\right.$$

### 2.2. The Equation of TEHL

The analytical equations for line contact TEHL are as follows: the Reynolds equation, the oil film thickness equation, the viscosity-pressure-temperature equation, the density-pressure-temperature equation, the load balance equation and the oil film energy equation, and the thermal balance equation.

1. The Reynolds equation

The Hertz contact half-width is much smaller than the gear tooth width when the gears are meshing. Because of this, the gear tooth contact is considered a line contact. The one-dimensional Reynolds equation, in its simplified form, is used to describe the fluid between the tooth surfaces. This equation can be expressed as follows:

(5) $$\frac{d}{dx}(\frac{\rho }{\eta }h{\left(x,t\right)}^{3}\frac{dp(x,t)}{dx})=12u\frac{d(\rho h(x,t))}{dx}$$

where *p* and *h* represent the oil film pressure and thickness, respectively, *η* and *ρ* are the density and viscosity of the lubricant. *u* is the relative sliding speed, defined in Equation (3).

The boundary conditions for the Reynolds equation in Equation (5) are as follows:

(6) $$\left\{\begin{array}{l} p\left(x_{\mathrm{in}},t\right)=p\left(x_{\mathrm{out}},t\right)=0\\ \frac{dp\left(x_{out},t\right)}{dx}=0 \end{array}\right.$$

where *x_in_* and *x_out_* denote the location of the entrance and exit, respectively.

2. The oil film thickness equation

In the one-dimensional line contact TEHL model, the oil film thickness consists of the center film thickness *h*_0_(*t*) without deformation, the geometric gap, and the elastic deformation *δ*(*x*, *t*), which can be expressed as follows:

(7) $$h(x,t)=h_{0}(t)+\frac{x^{2}}{2R(t)}+\delta (x,t)$$

where *R*(*t*) is the equivalent radius of curvature, defined in Equation (3).

In the deformation region *x_in_* < *x* < *x_out_*, the pressure distribution and the elastic deformation of the gear tooth are considered. According to the elastic half-space theory, the elastic deformation *δ*(*x*, *t*) of two contacting bodies under normal pressure can be expressed as follows [8,9]:

(8) $$\delta (x,t)=-\frac{2}{\pi E}\int_{x_{\mathrm{in}}}^{x_{\mathrm{out}}}p(s,t)\ln{\left(x-s\right)}^{2}ds$$

3. The viscosity-pressure-temperature equation

The viscous properties of lubricating oil are governed by temperature and pressure; the viscosity-pressure-temperature correlation is expressed as follows:

(9) $$\eta (x,t)=\eta_{0}\exp\left\{\left(\ln\eta_{0}+9.67\right)\left[{\left(1+5.1\times 10^{-9}p(x,t)\right)}^{z_{0}}\times {\left(\frac{T(x,t)-138}{T_{0}-138}\right)}^{-s_{0}}-1\right]\right\}$$

4. The density-pressure-temperature equation

The density of lubricating oil, similar to its viscosity, is also dependent on temperature and pressure. A density-pressure-temperature relationship is employed to characterize this behavior, as given in Equation (10).

(10) $$\rho =\rho_{0}\left[1+\frac{0.6\times 10^{-9}p}{1+1.7\times 10^{-9}p}-0.00065\left(T-T_{0}\right)\right]$$

where *ρ*_0_ is the environmental density of the lubricating oil.

5. The force balance equation

The load balance equation is formed by the unit line load w and the oil film force in the contact area, expressed as follows:

(11) $$\int_{x_{\mathrm{in}}}^{x_{\mathrm{out}}}p\left(x,t\right)dx=w\left(t\right)$$

6. The oil film energy equation

The lubricant film is altered in energy due to compression, friction, heat transfer, and other factors during the lubrication process. The oil film energy equation can be expressed as follows:

(12) $$c_{p}\rho \left(u\frac{\partial T}{\partial x}-w\frac{\partial T}{\partial z}\right)=k\frac{\partial^{2}T}{\partial z^{2}}-\frac{T}{\rho }\frac{\partial \rho }{\partial T}\left(u\frac{\partial p}{\partial x}\right)+\eta {\left(\frac{\partial u}{\partial z}\right)}^{2}$$

where *c_p_* and *k* are the specific heat capacity at constant pressure and heat transfer coefficient of the lubricant, respectively. Due to the $\partial^{2}T/\partial z^{2}$ term in Equation (12), it is necessary to give the temperature conditions of the lubricating oil at the upper and lower interfaces, expressed as follows:

(13) $$\left\{\begin{array}{l} T(x,0)=\frac{k}{\sqrt{\pi \rho_{1}c_{1}k_{1}u_{1}}}{\int_{-\infty }^{x}\left.\frac{\partial T}{\partial z}\right|}_{x,0}\frac{ds}{\sqrt{x-s}}+T_{0}\\ T(x,h)=\frac{k}{\sqrt{\pi \rho_{2}c_{2}k_{2}u_{2}}}{\int_{-\infty }^{x}\left.\frac{\partial T}{\partial z}\right|}_{x,h}\frac{ds}{\sqrt{x-s}}+T_{0} \end{array}\right.$$

where *T*_0_ is the initial temperature [9].

### 2.3. Surrogate Model for Solving TEHL

**Figure 2:**
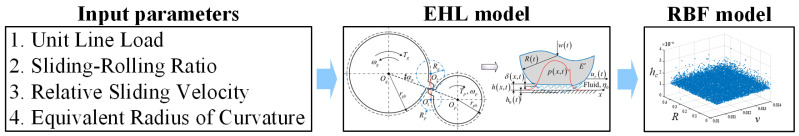
The solution scheme of the proposed method.

The direct coupling of the TEHL model with the dynamics model significantly reduces computational efficiency due to the slow iterative solution process of the TEHL model [17]. To address this limitation, the TEHL model is replaced by a surrogate model in this study. The computational flow of the TEHL agent model is illustrated in Figure 2.

In the meshing process, both the central film thickness and friction force critically influence tooth deformation and meshing stiffness. These parameters are determined by the unit line load, sliding-rolling ratio, relative sliding speed, and equivalent radius of curvature. To quantify these effects, the parameter variation ranges are extracted from the planetary gear dynamics model. Latin hypercube sampling is then employed to construct the design of experiments matrix, which is processed by the TEHL model to compute the film thickness and friction force. Subsequently, the response surface methodology (RSM) fits the input-output data, and the polynomial regression equation for the TEHL model is obtained as follows:

(14) $$\begin{array}{l} \left[h_{c}(t),\;f(t)\right]=c_{1}R_{1}(t)+c_{2}SR_{2}(t)+c_{3}V_{3}(t)+c_{4}W_{4}(t)+c_{5}R_{1}^{2}(t)+c_{6}SR_{2}^{2}(t)\\ \;\;\;\;\;\;+c_{7}V_{3}^{2}(t)+c_{8}W_{4}^{2}(t)+c_{9}R_{1}^{3}(t)+c_{10}SR_{2}^{3}(t)+c_{11}V_{3}^{3}(t)+c_{12}W_{4}^{3}(t)\\ \;\;\;\;\;\;+c_{13}R_{1}^{4}(t)+c_{14}SR_{2}^{4}(t)+c_{15}V_{3}^{4}(t)+c_{16}W_{4}^{4}(t)+c_{17}R_{1}(t)SR_{2}(t)+c_{18}R_{1}(t)V_{3}(t)\\ \;\;\;\;\;\;+c_{19}R_{1}(t)W_{4}(t)+c_{20}SR_{2}(t)V_{3}(t)+c_{21}SR_{2}(t)W_{4}(t)+c_{22}V_{3}(t)W_{4}(t)+c_{23} \end{array}$$

where *h_c_*(*t*), *f*(*t*) are the center oil film thickness and friction force, *c*_i_ (*i* = 1, ..., 23) are the polynomial coefficients.

In this study, R^2^ (R-squared) and RMSE (Root mean square error) are adopted as evaluation metrics to validate the surrogate model accuracy. As shown in Table 1, the surrogate model exhibits high computational precision, meeting the requirements for replacing the TEHL model.

## 3. Tribo-Dynamic Model for Dual-Star Planetary Gear Systems

In planetary gear systems, the planet gears not only rotate about their own axes but also revolve around the sun gear, resulting in complex kinematic relationships among components. By transforming the planetary gear train into an equivalent fixed-axis gear train, this intricate compound motion can be decomposed into multiple simple motions, thereby facilitating a clearer description of relative motions between components. Therefore, prior to establishing a lumped-parameter model (LPM) for planetary gear transmissions, it is necessary to perform coordinate transformation, which enables the derivation of more simplified and unified dynamic equations.

### 3.1. Coordinate Transformation of Dual-Star Planetary Gear Systems

**Figure 3:**
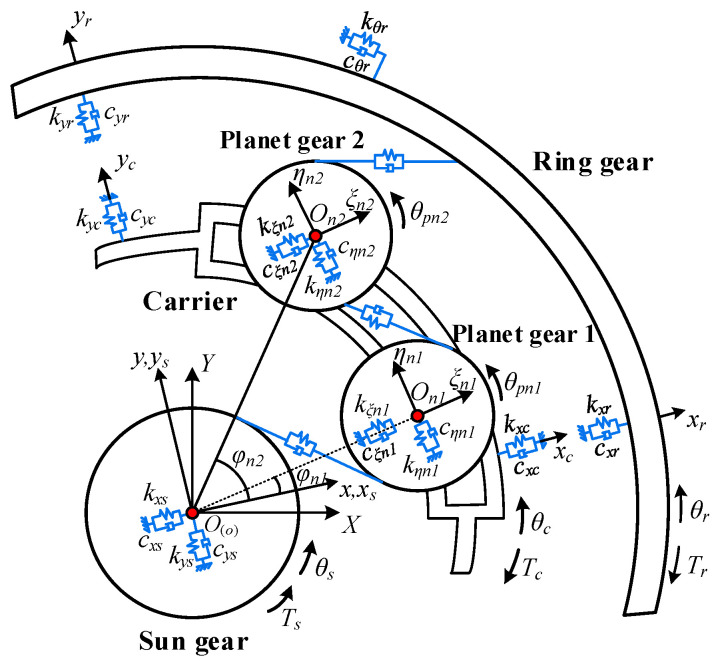
Dynamic model of dual-star planetary gear train.

Before transforming the coordinates, it is essential to establish the coordinate systems for the dual-star planetary gear train as illustrated in Figure 3. It includes three coordinate systems: (1) The stationary coordinate system *OXY*, with its origin located at the theoretical center of the sun gear. (2) The rotating coordinate system *oxy*, whose coordinate origin coincides with the theoretical center of the sun gear, but synchronously rotates with the planet carrier. (3) The rotating coordinate system *o_ni_ξ_ni_η_ni_* (where *n* denotes the *nth* planetary gear set and *i* = 1, 2), which also rotate synchronously with the planet carrier, its coordinate origin is located in the theoretical center of the *i*th planet gear, with the *ξ_n_*_1_ axis aligned along the connection line between the first planet gear and the sun gear. In order to facilitate the establishment of the coordinate system and the subsequent derivation of the dynamic equations, inside and outside of the two planet gears, the direction of the coordinate system is set to the same direction; the relevant parameters can be found in Ref. [2].

**Figure 4:**
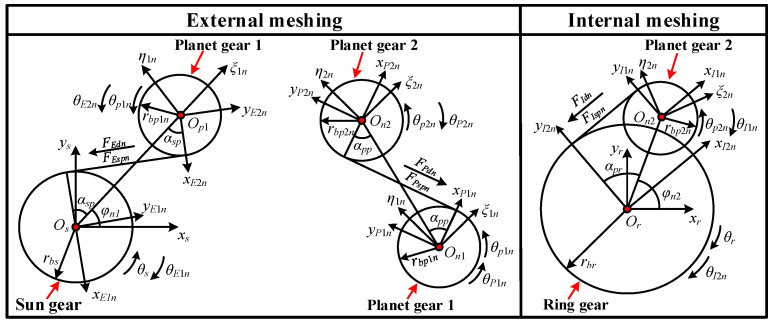
Fixed-axis gear train after transformation of dual-star planetary gear train.

As illustrated in Figure 4, the dual-star planetary gear train is transformed into a fixed-axis gear train. *x_Ain_*, *y_Ain_*, *θ_Ain_* (*A* = *E*, *I*, *P*; *i* = 1, 2) represent the *x*-directional translational displacement, *y*-directional translational displacement, and angular displacement about the *z*-axis of the *i*th gear in the *n*th set of transformed gear pairs, where *A* = *E*, *I*, *P* denotes sun-planet external meshing, planet-ring internal meshing, and planet-planet external meshing, respectively; *ξ_in_*, *η_in_* denote the translational displacements along *ξ* and *η* directions of the *i*th planet gear in the *n*th planetary gear set, where *i* = 1 represents the inner planet gear and *i* = 2 represents the outer planet gear; *F_Aspn_*, *F_Adn_* are the meshing forces and damping forces of the *n*th set of gear pairs.

Based on the geometric relationship between the rotating coordinate systems and fixed-axis gear train, the transformation relationships of the three gear meshing pairs are given in Equations (15)–(17).

(15) $$\left\{\begin{array}{l} x_{E1n}=-x_{s}\cos(\varphi_{n1}+\alpha_{sp})-y_{s}\sin(\varphi_{n1}+\alpha_{sp})\\ y_{E1n}=x_{s}\sin(\varphi_{n1}+\alpha_{sp})-y_{s}\cos(\varphi_{n1}+\alpha_{sp})\\ \theta_{E1n}=-\theta_{s}\\ x_{E2n}=-\xi_{n1}\cos(\alpha_{sp})-\eta_{n1}\sin(\alpha_{sp})\\ y_{E2n}=\xi_{n1}\sin(\alpha_{sp})-\eta_{n1}\cos(\alpha_{sp})\\ \theta_{E2n}=\theta_{p1n} \end{array}\right.$$

(16) $$\left\{\begin{array}{l} x_{I1n}=-\xi_{2n}\cos(\alpha +\alpha_{pr})-\eta_{2n}\sin(\alpha +\alpha_{pr})\\ y_{I1n}=\xi_{2n}\sin(\alpha +\alpha_{pr})-\eta_{2n}\cos(\alpha +\alpha_{pr})\\ \theta_{E1n}=-\theta_{p2n}\\ x_{I2n}=-x_{r}\cos(\varphi_{n2}+\alpha_{pr})-y_{r}\sin(\varphi_{n2}+\alpha_{pr})\\ y_{I2n}=x_{r}\sin(\varphi_{n2}+\alpha_{pr})-y_{r}\cos(\varphi_{n2}+\alpha_{pr})\\ \theta_{I2n}=-\theta_{r} \end{array}\right.$$

(17) $$\left\{\begin{array}{l} x_{P1n}=\xi_{1n}\cos(\pi -\gamma -\alpha_{pp})+\eta_{1n}\sin(\pi -\gamma -\alpha_{pr})\\ y_{P1n}=-\xi_{1n}\sin(\pi -\gamma -\alpha_{pr})+\eta_{1n}\cos(\pi -\gamma -\alpha_{pr})\\ \theta_{P1n}=\theta_{p1n}\\ x_{P2n}=\xi_{2n}\cos(\pi -\gamma -\alpha_{pr})+\eta_{2n}\sin(\pi -\gamma -\alpha_{pr})\\ y_{P2n}=-\xi_{2n}\sin(\pi -\gamma -\alpha_{pr})+\eta_{2n}\cos(\pi -\gamma -\alpha_{pr})\\ \theta_{P2n}=-\theta_{p2n} \end{array}\right.$$

where *α_sp_*, *α_pp_*, and *α_pr_* are the mesh angles of the sun-planet mesh pair, planet-planet mesh pair, and planet-ring mesh pair, respectively; *φ_n_*_1_ and *φ_n_*_2_ represent the phase angles of the inner and outer planet gears; *α* and *γ* denote the relative position angles of the sun-planet gear and planet-planet gear.

### 3.2. Dynamic Modeling of Dual-Star Planetary Gear Systems

During the carrier rotation, considering the influence of the non-inertial system, the translational and rotational accelerations of the sun gear and planet gears in the rotating coordinate system, *oxy*, are expressed as follows:

(18) $$\left\{\begin{array}{l} \alpha_{s}=\left({\ddot{x}}_{s}-2{\dot{y}}_{s}{\dot{\theta }}_{c}-x_{s}{\left({\dot{\theta }}_{c}\right)}^{2}-y_{s}{\ddot{\theta }}_{c}\right)i+\left({\ddot{y}}_{s}+2{\dot{x}}_{s}{\dot{\theta }}_{c}-y_{s}{\left({\dot{\theta }}_{c}\right)}^{2}+x_{s}{\ddot{\theta }}_{c}\right)j\\ \alpha_{ts}=\left({\ddot{\theta }}_{s}+{\ddot{\theta }}_{c}\right)k\\ \alpha_{pni}=\left({\ddot{\xi }}_{ni}-2{\dot{\eta }}_{ni}{\dot{\theta }}_{c}-(r_{ci}\cos(\varphi_{i}-\varphi_{1})+\xi_{ni}){\left({\dot{\theta }}_{c}\right)}^{2}-\eta_{ni}{\ddot{\theta }}_{c}\right)i_{pni}\\ \;\;\;+\left({\ddot{\eta }}_{ni}+2{\dot{\xi }}_{ni}{\dot{\theta }}_{c}-\eta_{ni}{\left({\dot{\theta }}_{c}\right)}^{2}+(r_{ci}\cos(\varphi_{i}-\varphi_{1})+\xi_{ni}){\ddot{\theta }}_{c}\right)j_{pni}\\ \alpha_{tpni}={\ddot{\theta }}_{pni}k_{pni} \end{array}\right.$$

where **α*_s_*** and **α*_ts_*** are the translational and rotational acceleration vectors of the sun gear, respectively; **α*_pni_*** and **α*_tpni_*** are the translational and rotational acceleration vectors of the planet gear, respectively; ${\dot{\theta }}_{c}$ is the carrier angular velocity in the stationary coordinate system; ${\ddot{x}}_{s}$ and ${\ddot{y}}_{s}$ denote the translational accelerations; $2{\dot{x}}_{s}{\dot{\theta }}_{c}$ and $2{\dot{y}}_{s}{\dot{\theta }}_{c}$ represent the Coriolis acceleration; $x_{s}{\left({\dot{\theta }}_{c}\right)}^{2}$ and $y_{s}{\left({\dot{\theta }}_{c}\right)}^{2}$ are the centripetal acceleration; $x_{s}{\ddot{\theta }}_{c}$ and $y_{s}{\ddot{\theta }}_{c}$ indicate the tangential acceleration; *r_ci_* is the distribution radius of the *i*th planet gear on the carrier.

Consequently, the dynamic model of the sun gear can be expressed as follows:

(19) $$\left\{\begin{array}{l} m_{s}({\ddot{x}}_{s}-2{\dot{y}}_{s}{\dot{\theta }}_{c}-x_{s}({\dot{\theta }}_{c}{)}^{2}-y_{s}{\ddot{\theta }}_{c})=-\sum_{n=1}^{N}F_{Espn}\sin(\varphi_{n1}+\alpha_{sp})-k_{xs}x_{s}-c_{xs}{\dot{x}}_{s}\\ m_{s}({\ddot{y}}_{s}+2{\dot{x}}_{s}{\dot{\theta }}_{c}-y_{s}({\dot{\theta }}_{c}{)}^{2}+x_{s}{\ddot{\theta }}_{c})=\sum_{n=1}^{N}F_{Espn}\cos(\varphi_{n1}+\alpha_{sp})-k_{ys}y_{s}-c_{ys}{\dot{y}}_{s}\\ J_{s}({\ddot{\theta }}_{s}+{\ddot{\theta }}_{c})=T_{s}+\sum_{n=1}^{N}(F_{Espn}+F_{Edn})r_{bs} \end{array}\right.$$

where *m_s_*, *J_s_*, and *r_bs_* represent the mass, moment of inertia, and base circle radius of the sun gear, respectively.

The dynamic model of the inner planet can be expressed as follows:

(20) $$\left\{\begin{array}{l} m_{pn1}({\ddot{\xi }}_{n1}-2{\dot{\eta }}_{n1}{\dot{\theta }}_{c}-(r_{c1}+\xi_{n1})({\dot{\theta }}_{c}{)}^{2}-\eta_{n1}{\ddot{\theta }}_{c})=F_{Espn}\sin(\alpha_{sp})-F_{Pspn}\sin(\pi -\gamma -\alpha_{pp})-k_{\xi n1}\delta_{pn1c\xi }-c_{\xi n1}{\dot{\delta }}_{pn1c\xi }\\ m_{pn1}({\ddot{\eta }}_{n1}+2{\dot{\xi }}_{n1}{\dot{\theta }}_{c}-\eta_{n1}({\dot{\theta }}_{c}{)}^{2}+(r_{c1}+\xi_{n1}){\ddot{\theta }}_{c})=-F_{Espn}\cos(\alpha_{sp})-F_{Pspn}\cos(\pi -\gamma -\alpha_{pp})-k_{\eta n1}\delta_{pn1c\eta }-c_{\eta n1}{\dot{\delta }}_{pn1c\eta }\\ J_{pn1}{\ddot{\theta }}_{pn1}=(F_{Espn}+F_{Edn})r_{bpn1}-(F_{Pspn}+F_{Pdn})r_{bpn1} \end{array}\right.$$

where *m_pn_*_1_, *J_pn_*_1_, and *r_bpn_*_1_ represent the mass, moment of inertia, and base circle radius of the *n*th inner planet, respectively.

The dynamic model of the outer planet can be expressed as follows:

(21) $$\left\{\begin{array}{l} m_{pn2}({\ddot{\xi }}_{n2}-2{\dot{\eta }}_{n2}{\dot{\theta }}_{c}-(r_{\xi 2}+\xi_{n2})({\dot{\theta }}_{c}{)}^{2}-(\eta_{n2}+r_{\eta 2}){\ddot{\theta }}_{c})=-F_{Pspn}\sin(\pi -\gamma -\alpha_{sp})-F_{Ispn}\sin(\alpha_{pr}+\alpha )-k_{\xi n2}\delta_{pn2c\xi }-c_{\xi n2}{\dot{\delta }}_{pn2c\xi }\\ m_{pn2}({\ddot{\eta }}_{n2}-2{\dot{\xi }}_{n2}{\dot{\theta }}_{c}-(\eta_{n2}+r_{\eta 2})({\dot{\theta }}_{c}{)}^{2}+(r_{\xi 2}+\xi_{n2}){\ddot{\theta }}_{c})=F_{Pspn}\cos(\pi -\gamma -\alpha_{sp})+F_{Ispn}\cos(\alpha_{pr}+\alpha )-k_{\eta n2}\delta_{pn2c\eta }-c_{\eta n2}{\dot{\delta }}_{pn2c\eta }\\ J_{pn2}{\ddot{\theta }}_{pn2}=-(F_{Pspn}+F_{Pdn})r_{bpn2}+(F_{Ispn}+F_{Idn})r_{bpn2} \end{array}\right.$$

where *m_pn_*_2_, *J_pn_*_2_, and *r_bpn_*_2_ represent the mass, moment of inertia, and base circle radius of the *n*th outer planet, respectively. *r_ξ_*_2_ and *r_η_*_2_ are the components of the distribution radius *r_c_*_2_ of the outer planet on the planetary carrier in the ξ and η directions, respectively.

The dynamic model of the carrier can be expressed as follows:

(22) $$\left\{\begin{array}{l} m_{c}({\ddot{x}}_{c}-2{\dot{y}}_{c}{\dot{\theta }}_{c}-x_{c}({\dot{\theta }}_{c}{)}^{2}-y_{c}{\ddot{\theta }}_{c})=\sum_{n=1}^{N}(k_{\xi n1}\delta_{pn1c\xi }+c_{\xi n1}{\dot{\delta }}_{pn1c\xi })\cos(\varphi_{n1})+\sum_{n=1}^{N}\left(k_{\eta n2}\delta_{pn2c\eta }+c_{\eta n2}{\dot{\delta }}_{pn2c\eta }\right)\cos(\varphi_{n1})\\ \;\;\;\;\;\;\;\;\;\;\;\;-\sum_{n=1}^{N}\left(k_{\eta n1}\delta_{pn1c\eta }+c_{\eta n1}{\dot{\delta }}_{pn1c\eta }\right)\sin(\varphi_{n1})-\sum_{n=1}^{N}(k_{\xi n2}\delta_{pn2c\xi }+c_{\xi 2n}{\dot{\delta }}_{pn2c\xi })\sin(\varphi_{n1})-k_{xc}x_{c}-c_{xc}{\dot{x}}_{c}\\ m_{c}({\ddot{y}}_{c}+2{\dot{x}}_{c}{\dot{\theta }}_{c}-y_{c}({\dot{\theta }}_{c}{)}^{2}+x_{c}{\ddot{\theta }}_{c})=\sum_{n=1}^{N}(k_{\xi n1}\delta_{pn1c\xi }+c_{\xi n1}{\dot{\delta }}_{pn1c\xi })\sin(\varphi_{n1})+\sum_{n=1}^{N}(k_{\eta n2}\delta_{pn2c\eta }+c_{\eta n2}{\dot{\delta }}_{pn2c\eta })\sin(\varphi_{n1})\\ \;\;\;\;\;\;\;\;\;\;\;\;+\sum_{n=1}^{N}(k_{\xi n1}\delta_{pn1c\xi }+c_{\xi n1}{\dot{\delta }}_{pn1c\xi })\cos(\varphi_{n1})+\sum_{n=1}^{N}(k_{\eta n2}\delta_{pn2c\eta }+c_{\eta n2}{\dot{\delta }}_{pn2c\eta })\cos(\varphi_{n1})-k_{yc}y_{c}-c_{yc}{\dot{y}}_{c}\\ J_{c}{\ddot{\theta }}_{c}=\sum_{n=1}^{N}[(k_{\eta n1}\delta_{pn1c\eta }+c_{\eta n1}{\dot{\delta }}_{pn1c\eta })r_{c1}+(k_{\eta n2}\delta_{pn2c\eta }+c_{\eta n2}{\dot{\delta }}_{pn2c\eta })r_{c2}]-T_{c} \end{array}\right.$$

where *m_c_* and *J_c_* are the mass and moment of inertia of the carrier, *N* denotes the number of planetary gear sets. *r_c_*_1_ and *r_c_*_2_ represent the distribution radii of the inner and outer planet on the carrier, respectively.

Similarly, the dynamic model of the ring gear can be expressed as

(23) $$\left\{\begin{array}{l} m_{r}({\ddot{x}}_{r}-2{\dot{y}}_{r}{\dot{\theta }}_{c}-x_{r}({\dot{\theta }}_{c}{)}^{2}-y_{r}{\ddot{\theta }}_{c})=\sum_{n=1}^{N}F_{Ispn}\sin(\alpha +\varphi_{n}+\alpha_{pr})-k_{xr}x_{r}-c_{xr}{\dot{x}}_{r}\\ m_{r}({\ddot{y}}_{r}+2{\dot{x}}_{r}{\dot{\theta }}_{c}-y_{r}({\dot{\theta }}_{c}{)}^{2}+x_{r}{\ddot{\theta }}_{c})=-\sum_{n=1}^{N}F_{Ispn}\cos(\alpha +\varphi_{n}+\alpha_{pr})-k_{yr}y_{r}-c_{yr}{\dot{y}}_{r}\\ J_{r}({\ddot{\theta }}_{r}+{\ddot{\theta }}_{c})=-\sum_{n=1}^{N}(F_{Ispn}+F_{Idn})r_{br}-k_{\theta r}(\theta_{r}+\theta_{c})-c_{\theta r}({\dot{\theta }}_{r}+{\dot{\theta }}_{c}) \end{array}\right.$$

where *m_r_*, *J_r_*, and *r_br_* represent the mass, moment of inertia, and base circle radius of the ring gear, respectively.

In this dynamic model, *δ_pnicξ_* and *δ_pnicη_* represent the relative displacements between the *n*th planet (inner and outer planet gear) and the carrier in the *ξ_ni_* and *η_ni_* directions;

(24) $$\left\{\begin{array}{l} \delta_{pnic\xi }=\xi_{ni}-x_{c}\cos(\varphi_{n1})-y_{c}\sin(\varphi_{n1})\\ \delta_{pnic\eta }=\eta_{ni}+x_{c}\sin(\varphi_{n1})-y_{c}\cos(\varphi_{n1}) \end{array}\right.$$

where *φ_n_*_1_ is the phase angle of the inner planet gear; *x_c_* and *y_c_* are the translational displacements of the carrier along the *x* and *y* directions of the coordinate system *o_c_x_c_y_c_*.

**Figure 5:**
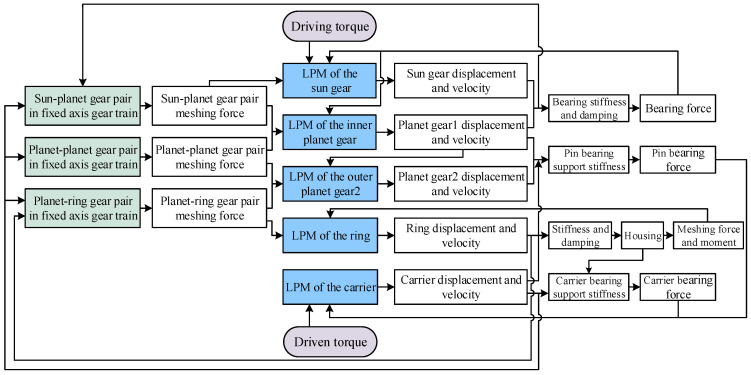
Dynamic model coupling flow chart of dual-star planetary gear train.

The dynamic modeling procedure for the dual-star planetary gear system is illustrated in Figure 5. The input torque drives the sun gear, and the respective displacements and velocities are obtained from the LPMs of the sun gear and the inner planet gear. Based on these, along with the displacement and velocity of the carrier, the meshing force between the sun gear and the inner planet gear is calculated using the fixed-axis gear train. Additionally, due to the bearing stiffness and damping, a bearing reaction force is generated. Both the meshing forces (sun-planet and inner planet) and the bearing force are then fed back into their respective LPMs for dynamic analysis. From the LPMs of the inner and outer planet gears, their respective displacements and velocities are obtained. Combined with the displacement and velocity of the carrier, the meshing force between the inner and outer planet gears is calculated using the fixed-axis gear train and then fed back into their LPMs. Similarly, the displacement and velocity of the ring gear are derived from its LPM. Along with the displacement and velocity of the carrier, the meshing forces between the outer planet gear and the ring gear are computed via the fixed-axis gear train and fed back into the LPMs of the outer planet gear and the ring gear, respectively. Additionally, since the ring gear is fixed, the housing applies a reaction force and moment to the LPM of the ring gear due to the effects of the stiffness and damping. The bearing force of the pin shaft is generated by the displacements and velocities of the carrier and the inner/outer planet gears acting on it. This force, along with the output load torque, is then fed back into the LPM of the carrier. In summary, the dynamic coupling among all components of the dual-star planetary gear system is established, completing the establishment of the dynamic model.

### 3.3. Tribo-Dynamic Coupling in Dual-Star Planetary Gear Systems

In order to analyze the interrelationship between the lubrication characteristics and the dynamic behavior of the planetary gear transmission, it is necessary to consider the effect of lubrication on the TVMS and backlash [17]. Based on this foundation, a tribo-dynamic analysis model for planetary gear transmissions can be established.

#### 3.3.1. Calculation of Oil Film Stiffness

Under lubricated conditions, the TVMS is governed by three stiffness components: the oil film stiffness, tooth stiffness, and foundation stiffness. While Refs. [7,29] provide complete formulations for tooth stiffness and foundation stiffness; this study only focuses on deriving the oil film stiffness and establishing the TVMS model incorporating lubricant film effects.

**Figure 6:**
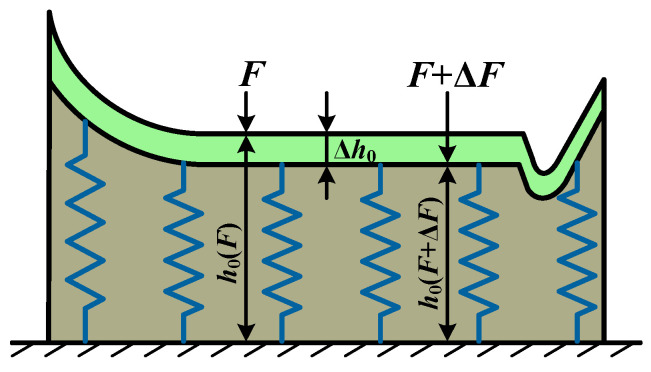
EHL contact stiffness equivalent spring model.

The equivalent model of oil film stiffness is illustrated in Figure 6. The central film thickness and elastic deformation at each meshing position are obtained through a surrogate model and dynamic simulation, respectively. The difference between the center oil film thickness and the elastic deformation represents the rigid body displacement. Under the action force *F*, the displacement of the rigid body is calculated as *h*_0_(*F*). Subsequent application of force increment Δ*F*, the displacement of the rigid body is *h*_0_(*F* + Δ*F*).

The oil film stiffness, *k_oil_,* can be calculated using the local slope method and is expressed as follows:

(25) $$k_{oil}(F,t)=\frac{\Delta F}{\Delta h_{0}}=\frac{\Delta F}{\left|h_{0}(F,t)-h_{0}(F+\Delta F,t)\right|}$$

Considering the EHL condition, the TVMS of the gear is calculated as follows:

(26) $$k_{mesh}=\sum_{i=1}^{m}k_{i},k_{i}=\frac{1}{\frac{1}{k_{oil}}+\frac{1}{k_{t}}+\frac{1}{k_{f}}}$$

where *m* denotes that there are *m* pairs of gear teeth currently in mesh; *k_t_* denotes the tooth stiffness, and *k_f_* denotes the foundation stiffness.

#### 3.3.2. Calculation of Backlash

**Figure 7:**
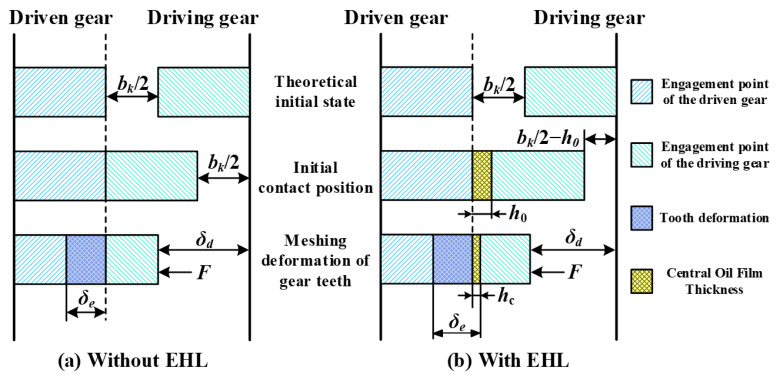
Diagram of gear meshing under lubrication.

To compensate for manufacturing errors, thermal expansion, and to prevent seizing due to poor lubrication, backlash is incorporated during gear design and assembly [30], as illustrated in Figure 7.

Under unlubricated conditions, gear pairs generate meshing forces only after contact occurs. At this point, the total tooth deformation *δₑ* can be expressed as follows:

(27) $$\delta_{e}=\left\{\begin{array}{l} \delta d-b_{k}/2,\;\;\;\delta_{d}>b_{k}/2\\ 0,\;\;\;\;\;\;-b_{k}/2\leq \delta_{d}\leq \;b_{k}/2\\ \delta d+b_{k}/2,\;\;\;\delta_{d}<-b_{k}/2 \end{array}\right.$$

where *δ_d_* is the normal compression deformation, and *bₖ* is the backlash.

However, under lubricated conditions, the tooth surfaces are separated by an oil film, and when the interfacial separation becomes smaller than the maximum central film thickness *h*_0_, hydrodynamic forces are generated between the gear pair. As the surfaces further approach each other, both the contact force and meshing deformation progressively increase. Under this condition, the total tooth deformation *δ_e_* can be expressed as follows:

(28) $$\delta_{e}=\left\{\begin{array}{l} \delta d-b_{k}/2+hc(t),\;\;\;\delta_{d}>b_{k}/2-hc(t)\\ 0,\;\;\;\;\;\;\;\;\;\;-b_{k}/2+hc(t)\leq \delta_{d}\leq \;b_{k}/2-hc(t)\\ \delta d+b_{k}/2-hc(t),\;\;\;\delta_{d}<-b_{k}/2+hc(t) \end{array}\right.$$

where *b_k_*/2 − *h_c_*(*t*) is the time-varying is the time-varying backlash under the EHL condition.

#### 3.3.3. Coupling Relationship Between Lubrication Model and Dynamic Model

**Figure 8:**
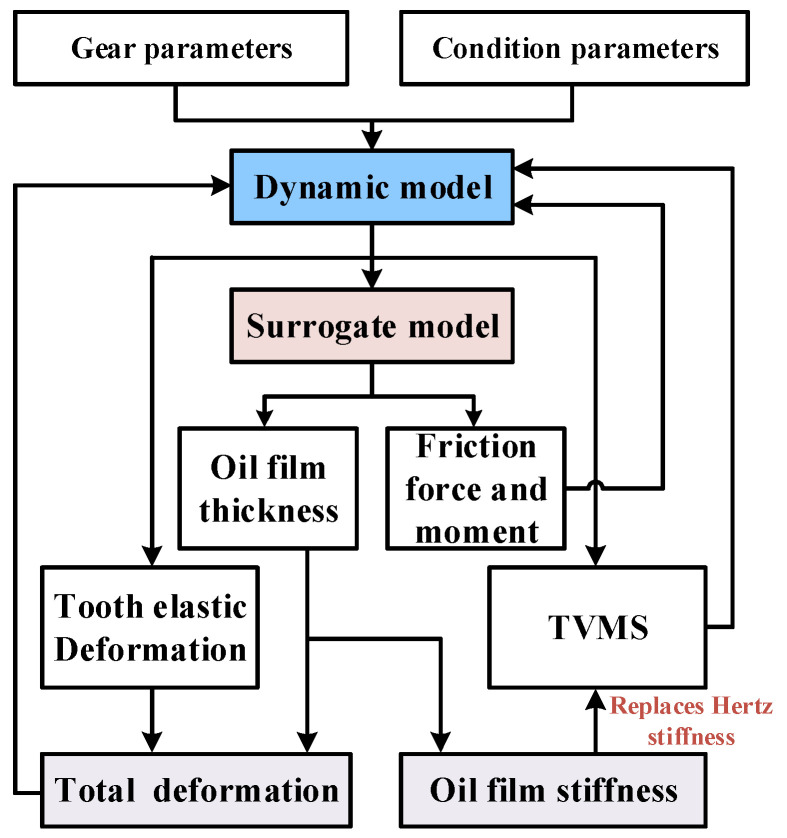
Flowchart of tribo-dynamic modeling.

In summary, the traditional TVMS and constant backlash are replaced by TVMS and time-varying backlash under EHL conditions, thereby establishing full coupling between the gear dynamics model and TEHL model. The computational workflow of the proposed model is illustrated in Figure 8.

First, the gear parameters and operational conditions are input into the planetary gear dynamics model to obtain dynamic parameters (e.g., meshing force, equivalent radius of curvature, relative sliding velocity, and sliding-rolling ratio). The surrogate model subsequently computes lubrication parameters—including central film thickness, friction force, and friction torque—based on these dynamic outputs. The total tooth deformation is then calculated considering lubrication effects, while the Hertz contact stiffness in the TVMS is replaced by oil film stiffness. These updated parameters, together with the friction force and torque, are fed back into the planetary gear dynamics model for iterative computation. Ultimately, the coupling relationship between the TEHL model and the planetary gear dynamics model is established.

## 4. Tribo-Dynamic Analysis of Dual-Star Planetary Gear Systems

By programming the established tribo-dynamics model of dual-star planetary gear systems in MATLAB software and performing simulations using SIMULINK, this paper analyzes the lubrication characteristics of the dual-star gear system and the influence mechanism of input speed and torque on the tribo-dynamics of the transmission system. All simulation results in this section are plotted using MATLAB software.

### 4.1. Lubrication Characterization Analysis of Dual-Star Gear Systems

To investigate the effects of relative sliding velocity *u_s_* and unit line load *w* on TEHL characteristics of the dual-star gear system, two control groups are designed: Relative sliding velocity *u_s_*: 2 m/s, 4 m/s, and 6 m/s; Unit line load *w*: 10^5^ N/m, 2 × 10^5^ N/m, and 3 × 10^5^ N/m. Based on these parameter combinations, the static lubrication characteristics of the tooth surface are analyzed.

**Figure 9:**
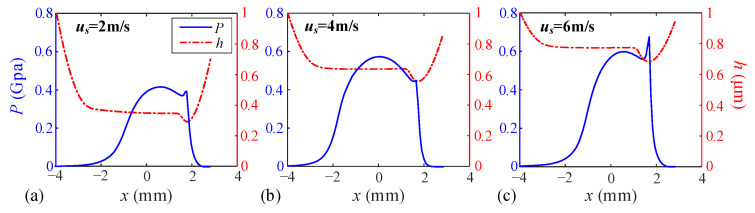
Distribution diagram of oil pressure (*P*) and film thickness (*h*) at different speeds. (**a**) Relative sliding velocity *u_s_* = 2 m/s, (**b**) Relative sliding velocity *u_s_* = 4 m/s, (**c**) Relative sliding velocity *u_s_* = 6 m/s.

**Figure 10:**
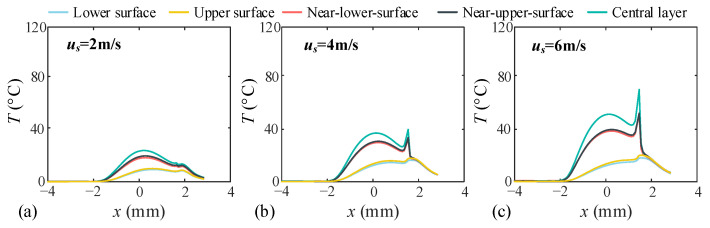
Temperature rise distribution of each layer of oil film at different speeds. (**a**) Relative sliding velocity *u_s_* = 2 m/s, (**b**) Relative sliding velocity *u_s_* = 4 m/s, (**c**) Relative sliding velocity *u_s_* = 6 m/s.

When the unit line load *w* is maintained at 10^5^ N/m, the variations in oil film pressure, film thickness, and temperature rise are investigated under varying relative sliding velocities *uₛ*, as illustrated in Figure 9 and Figure 10. The results demonstrate that increasing the sliding velocity significantly enhances the central oil film thickness, which progressed from 0.357 μm to 0.641 μm (+79.6%) and further to 0.788 μm (+120.7%). Correspondingly, the central oil film pressure exhibited growth from 0.421 GPa to 0.571 GPa (+35.6%) and 0.602 GPa (+42.9%). Regarding temperature distribution, the central layer exhibits the highest temperature rise, while adjacent layers show moderate increases, and surface layers remain stable.

**Figure 11:**
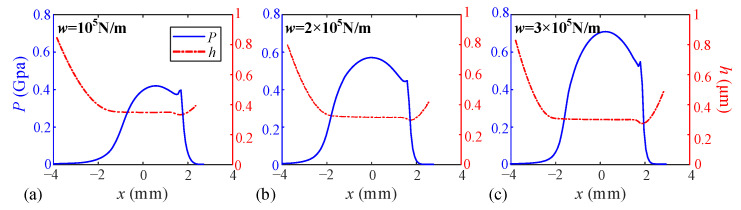
Distribution diagram of oil pressure (*P*) and film thickness (*h*) under unit line loads. (**a**) Unit line load *w* = 10^5^ N/m, (**b**) Unit line load *w* = 2 × 10^5^ N/m, (**c**) Unit line load *w* = 3 × 10^5^ N/m.

**Figure 12:**
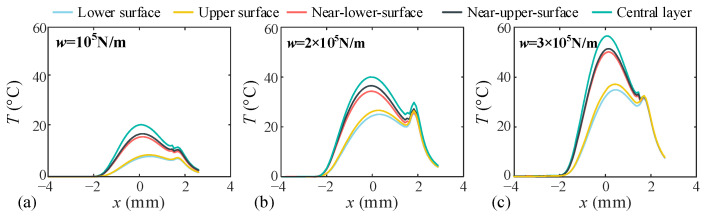
Temperature rise distribution of each layer of the oil film under different unit line loads. (**a**) Unit line load *w* = 10^5^ N/m, (**b**) Unit line load *w* = 2 × 10^5^ N/m, (**c**) Unit line load *w* = 3 × 10^5^ N/m.

When the relative sliding velocity *uₛ* is maintained at 2 m/s, the effects of unit line loads *w* are investigated, as shown in Figure 11 and Figure 12. The results demonstrate that increasing the unit line load significantly reduces the central film thickness, which decreases from 0.357 μm to 0.319 μm (−10.6%) and further to 0.292 μm (−18.2%). Correspondingly, the central oil film pressure exhibited substantial growth from 0.421 GPa to 0.571 GPa (+35.6%) and 0.692 GPa (+64.3%). Regarding temperature distribution, all layers displayed progressively intensified temperature rises with increasing line loads, showing uniformly enhanced thermal effects throughout the lubricant film structure.

### 4.2. Dynamic Analysis of Dual-Star Gear Systems

#### 4.2.1. Effects of Input Speed on the Dynamic Behavior of the System

**Figure 13:**
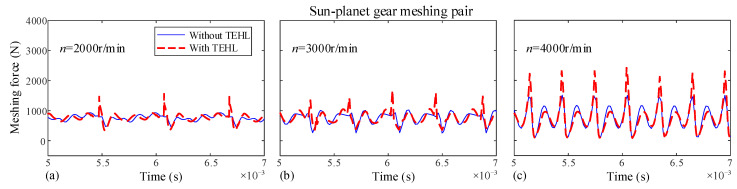
Dynamic meshing forces of the sun-planet gear at different input speeds. (**a**) Input speed *n* = 2000 rpm, (**b**) Input speed *n* = 3000 rpm, (**c**) Input speed *n* = 4000 rpm.

**Figure 14:**
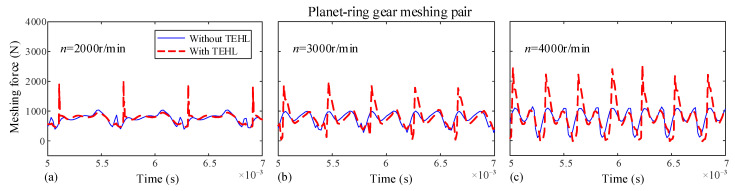
Dynamic meshing forces of the planet-ring gear at different input speeds. (**a**) Input speed *n* = 2000 rpm, (**b**) Input speed *n* = 3000 rpm, (**c**) Input speed *n* = 4000 rpm.

In this section, the dynamic characteristics of the dual-star gear system under different input speeds are investigated. Since both the planet-planet and sun-planet meshing pairs in this system exhibit similar external gear meshing characteristics, only the sun-planet and planet-ring meshing pairs are selected for analysis in this paper. The dynamic meshing forces for each meshing pair with and without TEHL are shown in Figure 13 and Figure 14, with an input torque *T* of 100 N·m and rotational speeds *n* of 2000, 3000, and 4000 r/min, respectively.

Under this condition, the dynamic meshing force fluctuates periodically around its mean value. At lower rotational speeds, the difference between TEHL and non-TEHL dynamic meshing forces is negligible. However, with increasing rotational speed, the TEHL dynamic meshing force exhibits stronger fluctuations and pronounced spikes. This is because an oil film forms between the meshing tooth surfaces when the gap between two tooth surfaces is less than the maximum central film thickness, *h_c_*_0_, lubricant squeeze generates a force that causes premature gear meshing. Consequently, this early meshing under lubrication leads to significantly higher impact forces compared to conditions without lubrication. Moreover, increasing rotational speeds further elevate the lubricant film thickness, thereby exacerbating meshing impacts.

#### 4.2.2. Effects of Input Torque on the Dynamic Behavior of the System

**Figure 15:**
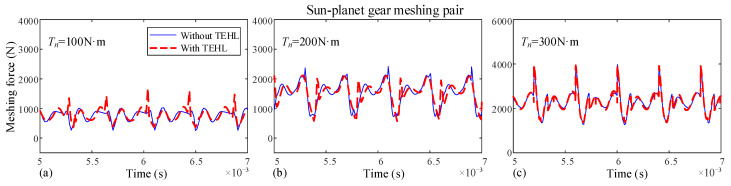
Dynamic meshing forces of the sun-planet gear at different input speeds. (**a**) Input torque *T_n_* = 100 N, (**b**) Input torque *T_n_* = 200 N, (**c**) Input torque *T_n_* = 300 N.

**Figure 16:**
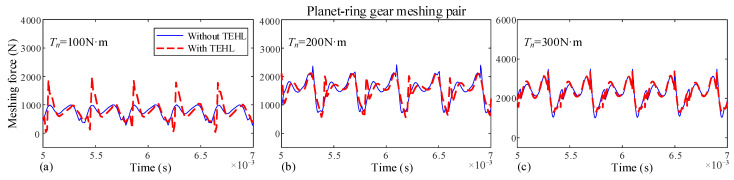
Dynamic meshing forces of the planet-ring gear at different input speeds. (**a**) Input torque *T_n_* = 100 N, (**b**) Input torque *T_n_* = 200 N, (**c**) Input torque *T_n_* = 300 N.

In this section, the dynamic characteristics of the dual-star gear system under different input torque conditions are investigated. The dynamic meshing forces for each meshing pair with and without TEHL are shown in Figure 15 and Figure 16, with a rotational speed *n* of 3000 r/min and input torques *T* of 100 N·m, 200 N·m, and 300 N·m, respectively.

When the input torque of the sun gear increases. The difference between TEHL and non-TEHL dynamic meshing forces is decreased. This is because higher torque leads to greater gear tooth deformation, making the influence of tooth deformation on dynamic meshing forces dominant while reducing the impact of oil film thickness.

**Figure 17:**
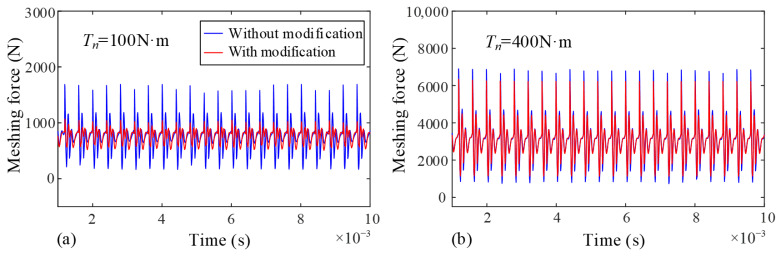
Dynamic meshing force comparison. (**a**) Input torque *T_n_* = 100 N, (**b**) Input torque *T_n_* = 400 N.

Figure 17 further demonstrates the effect of tooth deformation on dynamic meshing forces. Taking the sun-planet gear pair as an example, tooth profile modification is applied to compensate for deformation. The results show that under low input torque, the fluctuation of modified dynamic meshing forces is significantly reduced compared to the unmodified profiles. However, at high torque, the fluctuations of modified and unmodified meshing forces become nearly identical. This is because increasing torque exacerbates tooth deformation, leading to greater meshing impact and diminishing the effectiveness of profile modification in improving deformation. Meanwhile, the influence of oil film thickness further weakens, resulting in minimal differences in meshing force fluctuations between TEHL and non-TEHL conditions under high torque.

## 5. Experimental Verification of the Dual-Star Planetary Gear Tribo-Dynamic Model

**Figure 18:**
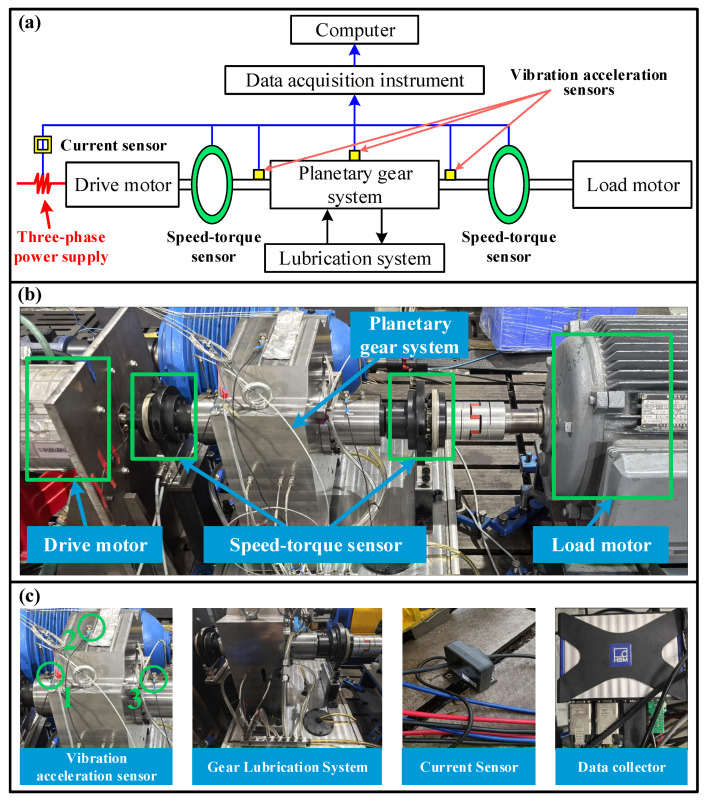
Schematic diagram and test bench for planetary gear system. (**a**) Schematic diagram of the test bench, (**b**) Physical test bench, (**c**) Key components of the test bench. Label “1”, “2”, and “3” represent the installation positions of three vibration acceleration sensors in the experiment.

To verify the influence of the TEHL characteristics on the dynamic response of planetary gear systems, a test bench for planetary gear transmission is designed, as shown in Figure 18. This test bench is composed of a drive motor, a load motor, a planetary gear system, a gear lubrication system, an HBM T40B (1000 N·m) speed-torque sensor, a DYTRAN vibration acceleration sensor, an AC/DC current sensor, and a Quantumx acquisition instrument. Among them, one HBM T40B (1000 N·m) speed-torque sensor is installed on the flange of the coupling connecting the output shaft of the drive motor and the sun gear, and the other sensor is installed on the flange of the coupling connecting the output shaft of the load motor and the planet carrier. Three DYTRAN vibration acceleration sensors are installed on the input shaft bearing, the ring surface, and the output shaft bearing, respectively. The A-phase current of the permanent magnet synchronous motor is measured by an AC/DC current sensor. The signals measured by each sensor are collected by the Quantumx acquisition instrument and saved in the computer. The sampling frequency of the test system is set to 19,200 Hz.

The experiments used Mobil ISO VG 32 synthetic gear oil (Mobil 32 XP) with a kinematic viscosity of 32 cSt at 40 °C, viscosity index ≥ 95, density 0.87 g/cm^3^, flash point 220 °C. The lubrication method of the gear system is oil injection lubrication. When the gears are in operation, the oil pump at the top of the housing starts to spray oil. Then, as the gears rotate, the oil flows back to the bottom of the housing and enters the oil pump to form an oil circuit. In the experiment, the on and off of the lubricating oil circuits of the gears and bearings can be controlled, respectively, to achieve the experiment under dry friction conditions and lubrication conditions.

### 5.1. Effects of Input Speed on the Vibration Behavior of the System

In order to explore the influence of lubrication and dry friction on the dynamic response of the system at different rotational speeds, the control parameters of the electric drive system are adjusted to make the average driving torque 25 N·m. Meanwhile, the motor speed is controlled within the range of 500 rpm to 3000 rpm, and the speed is changed by 500 rpm as the step size. Experimental measurements are conducted under conditions where the oil pump injects oil into and does not inject oil to the tooth surface.

**Figure 19:**
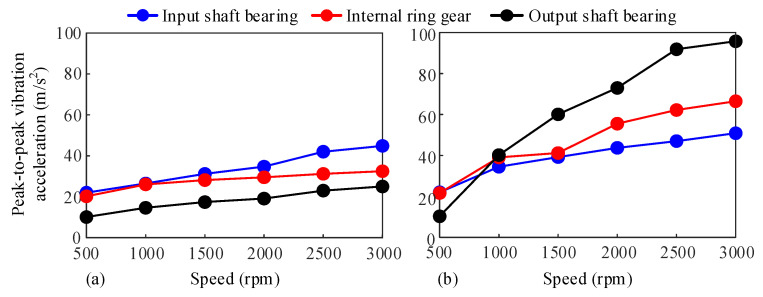
Peak-to-peak vibration acceleration of each measuring point at different input speeds. (**a**) Dry friction, (**b**) With lubrication.

Figure 19 presents the peak-peak vibration acceleration of each measurement point at different input speeds. It can be seen from the figure that the change in speed has a significant impact on the vibration acceleration of the system. As the speed increases, the peak-to-peak vibration acceleration at each measurement point gradually increases. Under dry friction conditions, the peak-peak vibration acceleration at the measurement point of the input shaft bearing is the largest. This is because the motor directly transmits the motor vibration to the input shaft through the coupling. Under lubrication conditions, the vibration acceleration of the system is significantly greater than that under dry friction conditions. And the higher the speed, the more obvious the difference between the two. At this time, the peak-peak vibration acceleration at the measurement point of the output shaft bearing is the largest. This is because under lubrication conditions, the transmission efficiency is improved, the actual torque transmitted at the output end is higher, and the load borne by the output shaft bearing is greater. In addition, under lubrication conditions, the lubricating oil forms a viscous damping layer on the tooth surface, absorbing the high-frequency vibration energy at the input end, so the peel-peak vibration acceleration at the measurement point of the output shaft bearing is the largest.

**Figure 20:**
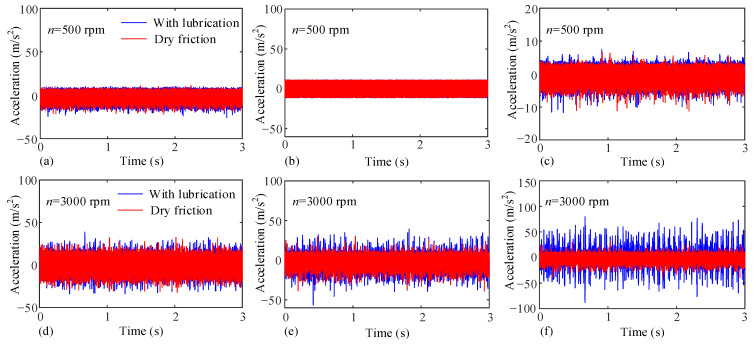
Measured vibration acceleration under different input speeds. 500 rpm: (**a**) Input shaft bearing, (**b**) Gear ring surface, (**c**) Output shaft bearing; 3000 rpm: (**d**) Input shaft bearing, (**e**) Gear ring surface, (**f**) Output shaft bearing.

The speeds are 500 rpm and 3000 rpm, respectively. The measurement results of each vibration sensor are presented in Figure 20. It can be seen that under the same torque, compared with the working condition of 500 rpm, the vibration acceleration results of lubrication and dry friction at 3000 rpm show a greater difference. The vibration acceleration fluctuation under lubrication conditions becomes larger and produces obvious peaks. This is related to the meshing impact caused by the oil film on one hand, and on the other hand, it is because the thickness of the oil film increases with speed. At high speeds, the oil film thickens, which greatly advances the position of the gear teeth at the meshing point, resulting in an increase in meshing impact. The meshing impact vibration is transmitted to the measurement points of each vibration sensor, causing the vibration acceleration to increase. The conclusion of this part is also consistent with that in Section 4.2.1.

### 5.2. Effects of Input Torque on the Vibration Behavior of the System

**Figure 21:**
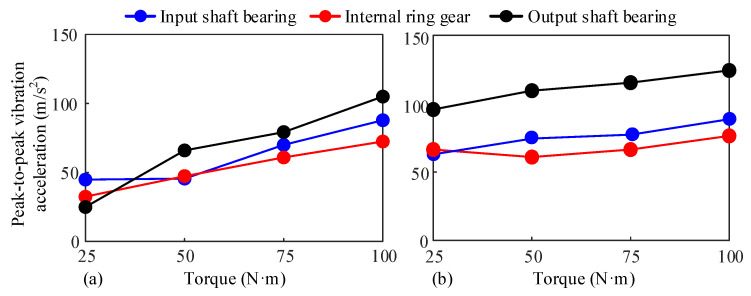
Peak-to-peak value of vibration acceleration of each measuring point under different input torques. (**a**) Dry friction, (**b**) With lubrication.

In order to explore the influence of lubrication and dry friction on the dynamic response of the system under different torques, the control parameters of the electric drive system are adjusted to keep the load speed constant at 776.8 rpm (corresponding to 3000 rpm at the drive end), the drive torque within the range of 25 N·m to 100 N·m, and the step is set to 25 N·m. Experimental measurements are conducted under conditions where the oil pump injects oil to the tooth surface and does not inject oil, respectively. Figure 21 presents the peak-to-peak vibration acceleration of each measurement point under different torques. The results show that the torque variation has a significant influence on the vibration acceleration of the system. As the torque increases, the peak-to-peak vibration acceleration at each measurement point gradually increases. Among them, the peak-to-peak vibration acceleration at the output shaft bearing is the largest, and its growth with the torque change is significantly greater than that at the input shaft bearing and the ring. This is because the reducer has the characteristic of deceleration and torque increase. The greater the average torque transmitted at the output and the torque fluctuation, the greater the load at the output shaft bearing. Therefore, the vibration acceleration of the output shaft bearing is the largest. In addition, under lubrication conditions, the vibration acceleration of the system is significantly greater than that under dry friction conditions, and the smaller the driving torque, the more obvious the difference between the two becomes.

**Figure 22:**
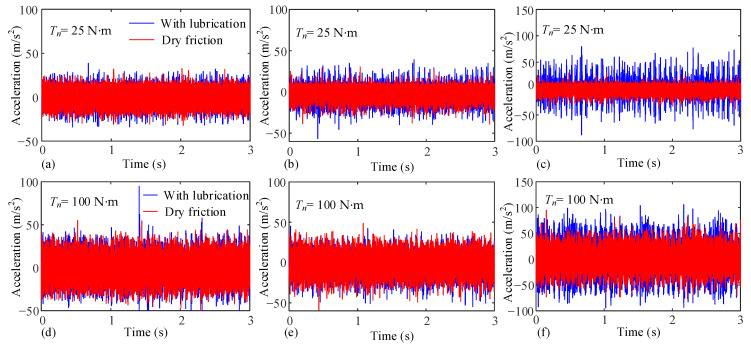
Measured vibration acceleration under different input torques. 25 N·m: (**a**) Input shaft bearing, (**b**) Gear ring surface, (**c**) Output shaft bearing; 100 N·m: (**d**) Input shaft bearing, (**e**) Gear ring surface, (**f**) Output shaft bearing.

The torques are 25 N·m and 100 N·m, respectively. The measurement results of each vibration sensor are presented in Figure 22. It can be observed that at the same rotational speed and torque, the vibration acceleration fluctuation under lubrication conditions is greater. Compared with the working condition of 100 N·m, the vibration acceleration results of lubrication and dry friction under the working condition of 25 N·m show more significant differences and produce more obvious peaks. This phenomenon occurs because when oil is sprayed onto the tooth surfaces, an oil film forms between the contacting surfaces. When the interfaces of the two teeth do not come into contact but the relative distance is less than the maximum central film thickness, the tooth pairs already exert force and undergo meshing deformation under the action of lubricating oil, causing the gear tooth pairs to enter the meshing state prematurely. The premature meshing of gear pairs increases the meshing deformation, thereby intensifying the meshing-in impact. Furthermore, as the film thickness increases with decreasing torque, the oil film becomes thicker at lower torque levels. This causes the position of the gear teeth at the meshing point to advance significantly, resulting in increased meshing impact. The meshing impact vibration is then transmitted to the measurement points of each vibration sensor, leading to increased vibration acceleration.

## 6. Conclusions

Considering the unclear coupling mechanism between TEHL and dynamic behaviors in planetary gear systems, a novel tribo-dynamic model of the dual-star planetary gear transmission system is proposed in this paper. The dynamic characteristics of the transmission system under different operating conditions are analyzed through simulation, and by establishing a test bench, its validity is experimentally verified. The main conclusions are as follows:

1. Regarding lubrication characteristics, increasing the relative sliding velocity significantly enhances both oil film thickness and pressure, while increasing unit line load reduces film thickness but substantially increases pressure. Furthermore, the relative sliding velocity primarily governs central lubricant layer temperature rise, whereas unit line load induces synchronous interfacial temperature increases across all layers.
2. Gear lubrication significantly influences the dynamic meshing characteristics of dual-planet gear systems. Under high-speed conditions, increased oil film thickness induces premature gear meshing, generating significant meshing impacts. Conversely, under high-torque conditions, tooth deformation becomes the dominant factor governing meshing force fluctuations, thereby reducing the influence of lubrication effects. Furthermore, while tooth profile modification effectively mitigates vibrations at low torque levels, its beneficial effects progressively diminish with increasing torque.
3. Experimental results demonstrate that lubrication significantly amplifies transmission system vibrations, particularly under high-speed or low-torque conditions. This phenomenon correlates with lubrication-induced variations in dynamic meshing forces, thereby validating the accuracy of the proposed model.

## Figures and Tables

**Table 1:** Accuracy test of the surrogate model.

Surrogate Model	R^2^	RSME
Sun-planet gear pair	0.9415	0.01634
Planet-ring gear pair	0.9523	0.01556
Planet-planet gear pair	0.9554	0.01509

## Data Availability

Data are contained within the article.
